# Untargeted serum metabolomics and air pollution in Parkinson’s disease

**DOI:** 10.21203/rs.3.rs-8857902/v1

**Published:** 2026-03-02

**Authors:** Dayoon Kwon, Kimberly C. Pual, Yuyuan Lin, Danielle Thordarson, Jun Wu, Jason Su, Dean P. Jones, Jeff M. Bronstein, Beate Ritz

**Affiliations:** Stanford University; University of California, Los Angeles; University of California, Los Angeles; University of California, Los Angeles; University of California, Irvine; University of California, Berkeley; Emory University; University of California, Los Angeles; University of California, Los Angeles

## Abstract

Mechanisms linking air pollution to Parkinson’s disease (PD) remain poorly understood despite consistent toxicological and epidemiological evidence. We investigated metabolic disturbances associated with air pollution in PD using untargeted metabolomics. Serum samples were collected from 616 PD patients and 271 controls residing in central California, a region with high air pollution. Exposures to particulate matter (PM_2.5_), carbon monoxide, and nitrogen dioxide were estimated for the 1-, 3-, and 5-year periods prior to serum collection using residential addresses and three modeling approaches: chemical transport, dispersion, and land use regression. We conducted a metabolome-wide association study using untargeted serum metabolomics based on liquid chromatography-high-resolution mass spectrometry (detecting 2,716 HILIC and 2,046 C18 features). Pathway enrichment analysis identified biological pathways associated with air pollution exposure in PD patients and controls. We compared our findings with 22 previous air pollution metabolomics studies to assess external validity. We identified 26 annotated metabolites and 23 metabolic pathways associated with air pollution exposure, particularly PM_2.5_ and traffic-related pollutants, in PD patients and controls. Metabolic profiles observed in controls aligned with prior studies, supporting external validity. Profiles in PD patients additionally indicated disease-specific disruptions. Air pollution was associated with inflammation-related lipid metabolism (e.g., increased leukotrienes; decreased eicosatrienoic acid and docosahexaenoic acids) and several amino acids (e.g., alanine, aspartate, and glutamate) in PD patients. We also found a reduction in tyrosine levels, possibly related to PD. Togethger, these findings suggest that air pollution may contribute to PD through inflammation, oxidative stress, and mitochondrial dysfunction.

## INTRODUCTION

Epidemiological evidence increasingly associates air pollution with Parkinson’s disease (PD), particularly long-term exposure to particulate matter (PM_2.5_), carbon monoxide (CO), and nitrogen dioxide (NO_2_), key markers indicative of traffic-related air pollution (TRAP).^1,2^ However, the biological pathways underlying these relationships remain largely unknown. Untargeted metabolomics, a powerful analytical technique capable of simultaneously profiling thousands of metabolites, has emerged as an important method for identifying exposure biomarkers and elucidating biological pathways affected by environmental exposures. Recent air pollution metabolomics studies consistently highlight perturbations in metabolic pathways linked to inflammation, oxidative stress, and energy metabolism.^3,4^ Similarly, PD metabolomics research has identified disruptions in lipid metabolism and amino acid pathways that are critical to brain health and mitochondrial function.^5^ Building upon these prior findings, we conducted a metabolome-wide association study (MWAS) within a case-control study located in central California, an area known for high levels of air pollution. This study aimed to (1) identify serum-measured metabolic disturbances associated with PM_2.5_ and TRAP exposure (measured by CO and NO_2_) among PD patients and controls, (2) compare findings with existing air pollution metabolomics studies, and (3) provide biological insights into mechanisms linking air pollution exposure to PD.

## METHODS

### Study population

We analyzed metabolomic profiles in 616 PD patients and 271 population-based controls from the Parkinson’s Environment and Gene (PEG) study, conducted in Fresno, Kern, and Tulare counties in central California. Participants were recruited in two waves: PEG1 (2000–2007) and PEG2 (2010–2017). All individuals with available serum samples for metabolomics analysis were included.

PD patients were eligible if they had resided in California for at least five years and had received a PD diagnosis within the past three (PEG1) or five years (PEG2), with an average time since diagnosis of 3 years (SD = 2.6). Recruitment sources included local clinics, neurologists, medical groups, radio advertisements, and the California PD registry. All participants underwent in-person neurologic examinations conducted by University of California, Los Angeles (UCLA) movement disorder specialists, who confirmed idiopathic PD based on clinical characteristics.

Controls were randomly selected from Medicare rolls or property tax assessor records listings of residential parcels. Additional inclusion criteria for controls included being at least 35 years of age, having resided in California for five years or more, currently residing in one of the three counties, and not having received a diagnosis of Parkinsonism. Further details about recruitment procedures have been published elsewhere.^6^

The study was conducted in accordance with the Declaration of Helsinki and all methods were carried out in accordance with relevant guidelines and regulations. The study protocol was approved by the UCLA Institutational Review Baord. Written information consent was obtained from all participants priror to participation.

#### Air pollution exposure assessment

We used established air pollution models to estimate annual average pollutant concentrations at participants’ geocoded residential locations for each year up to the year of PD diagnosis for cases or the interview year for controls (latest possible year was 2016). We focused our exposure assessment on the period from 1996 to 2016 to align with the timeframe of serum sample collection for metabolomics profiling.

The main exposures in this study were PM_2.5_, modeled using a chemical transport model (CTM), and local TRAP, measured by CO using the California Line Source Dispersion Model, version 4 (CALINE4). For PM_2.5_, we applied a fine-resolution geospatial model providing validated 1 km resolution exposure estimates across North America.^7^ This model integrates GEOS-Chem CTM outputs, satellite aerosol optical depth (AOD) observations, and ground-based PM_2.5_ measurements using geographically weighted regression. For local TRAP, the CALINE4 dispersion model estimated CO concentrations by summing up vehible exhaust emissions dispersed from roadway links within 1.5 km of geocoded residential addresses using local traffic and meteorological data.^8,9^

We also examined additional exposures, including PM_2.5_ and regional TRAP, measured by NO_2_, both modeled using a land use regression (LUR) approach for the same 1996–2016 period.^10^ PM_2.5_ estimates were derived from fixed-site government-regulatory monitoring data and Google Streetcar data, while NO_2_ estimates additionally incorporated research-based saturation observations. The LUR models provided 100 m spatial resolution and incorporated inputs such as daily vehicle miles traveled, remote sensing data (AOD and NO_2_), biweekly vegetation indices, daily weather data, and land-use/land-cover features.

For brevity, we refer to pollutants in the format *pollutant (modeling approach)* throughout the text – for example, PM_2.5_ (CTM), CO (CALINE4), PM_2.5_ (LUR), and NO_2_ (LUR). In this study, the CTM- and CALINE4-based models were prioritized based on their stronger validation performance (R^2^ = 0.86 for PM_2.5_ [CTM] and 0.92 CO [CALINE4]) compared with the LUR-based models (R^2^ = 0.65 for PM_2.5_ [LUR] and 0.84 for NO_2_ [LUR]),^7,10,11^ and their prior use in air pollution-PD epidemiologic studies conducted in California and globally.^2,12^

To explore different exposure periods, we calculated 1-year, 3-year, and 5-year average exposures using the calendar years preceding the year of serum sample collection and examined per interquartile range (IQR) increases. Since serum sample collection began in 2001, the earliest exposure window analyzed was 1996–2001 for the 5-year average. Participants for whom we had data available for at least 60% of the exposure period were included in the analysis, and any remaining missing data (up to 40% of the period) were imputed by averaging concentrations from adjacent years (4 patients and 3 controls). A small proportion of participants (0.1–3%) had imputed values, and mean exposure levels were comparable between those with and without imputed data based on t-tests (p = 0.4–1.0), suggesting minimal exposure misclassification due to imputation.

#### High-resolution metabolomics (HRM)

Serum samples were collected and processed following previously published procedures.^13^ Detailed protocols for sample handling, preparation, and liquid chromatography-high resolution mass spectrometry (LC-HRMS) instrumentation are provided in the Supplemental Material. Briefly, sample preparation and data acquisition were conducted using a standard high-resolution metabolomics (HRM) workflow,^5^ with triplicate analysis of each sample using hydrophilic interaction chromatography (HILIC) with positive electrospray ionization (ESI) and C18 chromatography with negative ESI.

Quality control (QC) included NIST 1950 reference samples, analyzed at the beginning and end of each run,^14^ and pooled plasma QC samples, analyzed at the start, middle, and end of each batch for batch effect correction. Data quality was assessed using XCMS and internal standards, applying thresholds for mass accuracy (< 5 ppm), Pearson correlation within technical replicates (> 0.9), and feature intensity coefficient of variation (CV < 30%). Samples not meeting these criteria were reanalyzed. Further details on the QC workflow are provided in a prior publication.^5^

Samples were analyzed in two LC-HRMS runs. To ensure consistency, retention time adjustment and feature alignment were performed using the *apLCMS* R package,^15^ with *m/z* tolerance of 1e-05 and retention time tolerance of 38.246 seconds (HILIC) and 37.016 seconds (C18). After alignment, 2,919 features were identified for HILIC and 2,226 for C18.

To ensure data quality, we retained only metabolic features detected in > 50% of study samples, with a median CV < 30% among technical replicates and Pearson correlation > 0.9. This filtering resulted in 2,716 HILIC and 2,046 C18 features included in the analysis. Missing values were imputed using the lowest detected value for each feature, followed by log_2_ transformation, quantile normalization, and batch correction using ComBat. Principal component analysis identified technical variation across samples, and an additional Combat correction was applied using an indicator for clustering patterns to further reduce non-biological signals.^5^

### Statistical analysis

To identify metabolite features associated with air pollution exposure, we performed an untargeted metabolome-wide association study (MWAS) in PD patients using empirical Bayes linear regression models. The model estimated log_2_ fold changes (log_2_FC) per IQR increase in air pollution, adjusting for age, gender, race/ethnicity (white vs. non-white), education (years), sample collection year, and study wave (PEG1 vs. PEG2). We applied two strategies to identify statistically significant metabolite features: (1) We applied a false discovery rate (FDR) threshold of < 0.05 to correct for multiple comparisons; however, we were only able to annotate one metabolite feature that passed this threshold. (2) Given the exploratory nature of this analysis, we adopted a more pragmatic replication-based strategy: we considered features of interest if they met nominal statistical significance (p < 0.05) and were consistent in the direction of effect estimates in both PEG1 and PEG2. While using a raw p-value threshold may increase false positives, requiring consistent effect direction across PEG1 and PEG2 strengthens the credibility of findings. High correlations of effect estimates across study waves support the reproducibility of associations and help prioritize candidate metabolites for downstream analysis (Supplementary Fig. 1).

We additionally performed a full MWAS in control participants as an independent analysis. This allowed us to assess whether the associations observed in PD patients were also present in individuals without PD and to check whether control results in general aligned with prior findings from serum- or plasma-based air pollution-metabolomics studies. Comparing the two sets of results enabled us to explore potential disease-specific metabolic responses to air pollution exposure. We also repeated analyses using alternative exposure assessment models – PM_2.5_ and NO_2_ from LUR – to evaluate robustness across different air pollution modeling approaches.

Pathway analyses were performed using the *mummichog* approach in *MetaboAnalystR*,^16^ based on MWAS results derived from empirical Bayes linear regression models. Parameters included a significance threshold of p < 0.05, mass tolerance of 10 ppm, and mixed ion mode. Pathways were selected if they included ≥ 5 metabolites associated with air pollution exposure and showed overrepresentation with p < 0.05.

We annotated metabolite features associated with air pollutants in MWAS (p < 0.05, same direction in PEG1 and PEG2) that were also enriched in a metabolic pathway (p < 0.05). This approach helped minimize false positives while allowing the use of a less statistically conservative threshold for MWAS to guide exploratory analyses. Annotation was performed using three approaches: (1) features were matched to a database of authenticated chemical standards identified by the Emory laboratory using MS/MS and authentic standards, as these offer the highest annotation confidence.^17,18^ The error tolerance was set to 10 ppm for m/z and 30 seconds for retention time; (2) *mummichog* was used to predict functional activity and to annotate features based on metabolic pathways and networks using predicted ions and pathway associations;^19^ and (3) *xMSannotator* matched m/z values for adducts (positive/negative ESI mode) to the Human Metabolome Database with a 10 ppm mass error threshold, leveraging intensity and retention time correlations.^20^ Confidence scores (0–3) were assigned based on a multilevel scoring algorithm, and only annotations with a score ≥2 were included.

## RESULTS

### Participant characteristics

The mean age of PD patients in our study was 67 years at diagnosis and 70 years at time of serum sample collection, with an average PD duration of 3 years (Table 1). Most patients were men (63%), nonHispanic White (76%), and never-smokers (53%). Controls had a mean age of 66 years at the time of interview and serum collection. The control group was similarly composed of men (62%) and had similar education levels, but had a higher proportion of non-Hispanic White individuals (86%) and a greater percentage of current or former smokers (52%). Most controls with available biosamples participated in the PEG1 wave.

#### Air pollutant distributions and correlations

The mean estimated air pollutant exposure levels in the year prior to serum sample collection were 12.7 μg/m^3^ for PM_2.5_ (CTM) and 3.7 ppb for CO (CALINE4) (Supplementary Table 1). The exposure distributions were similar for longer averaging periods (e.g., 3 and 5 years prior). CO (CALINE4) was moderately correlated with PM_2.5_ (CTM) (ρ = 0.59–0.64) and with NO_2_ (LUR) (ρ = 0.62–0.64). These moderate correlations reflect differences in source coverage: CALINE4-based CO captures only local vehicle exhaust within 1.5 km of the residence, whereas PM_2.5_ (CTM) and NO_2_ (LUR) represent total concentrations from all sources, though NO_2_ more strongerly reflects traffic-related air pollution (Supplementary Fig. 2). PM_2.5_ (CTM) and PM_2.5_ (LUR) were also only moderately correlated (ρ = 0.49–0.57), highlighting differences between the CTM and CALINE4 models used for primary analyses and the LUR-based models used only for comparison.

#### Metabolome-wide association study (MWAS)

We first examined air pollution-metabolite associations only in PD patients using untargeted MWAS. For PM_2.5_ (CTM), numerous features were identified in C18 mode (FDR < 0.05: 5–14; p < 0.05 with replication: 221–255) and in HILIC (p < 0.05 with replication: 138–162) across 1-, 3-, 5-year exposure averages (Supplementary Table 2), though none of the FDR-significant features were annotatable. Among annotated metabolites meeting the nominal threshold (p < 0.05 with replication), we observed negative associations with inflammation-related lipids, including hydroxydocosahexaenoic acid (HDHA) and eicosatrienoic acid, particularly at longer-term exposures (e.g., 5-year HDHA: log_2_FC=−0.6, 95% CI=−1.2, −0.04). In contrast, leukotriene was positively associated with longer-term exposure (5-year: log_2_FC = 0.4, 95% CI = 0.02, 0.8; Fig. 1; Supplementary Data 1). Similarly, amino acids such as serine, glutamate, and aspartate, as well as the Vitamin A metabolite retinol, showed inverse associations that tended to strengthen with longer exposure averages, suggesting cumulative metabolic disruption.

For CO (CALINE4) in PD patients, we identified a larger number of features in HILIC (FDR < 0.05: 29–31; p < 0.05 with replication: 167–190) and C18 (p < 0.05 with replication: 42–46); dTDP was the only annotated metabolite passing the FDR < 0.05 across all three exposure averages (1-, 3-, and 5-year), consistently showing lower abundance with higher CO exposure (e.g., 1-year: log_2_FC=−0.1, 95% CI=−0.2, −0.07). Tyrosine was also nominally lower in the year closest to sample collection (1-year: log_2_FC=−0.08, 95% CI=−0.1, −0.02).

Using alternative exposure models to conduct the MWAS in PD patients, we found only one metabolite feature that met FDR < 0.05 for PM_2.5_ (LUR) in HILIC, and none for NO_2_ (LUR). However, several nominal associations for PM_2.5_ (LUR) were consistent in direction and magnitude with those observed using the CTM model, including HDHA (5-year: log_2_FC=−0.6, 95% CI=−1.0, −0.2) and serine (5-year: log_2_FC=−0.6, 95% CI=−1.1, −0.05).

In controls, only one metabolite in the C18 mode met the FDR < 0.05 threshold for CO (CALINE4, all exposure windows) and PM_2.5_ (LUR, 5-year) (Table S3); however, these features could not be annotated. As a result, comparisons between PD and controls were limited to metabolites with nominal statistical significance (p < 0.05 with replication; Table S4). Effect estimates were moderately to strongly correlated for PD patients and controls, particularly with the PM_2.5_ (LUR), suggesting shared metabolic responses to particulate exposure (Supplementary Fig. 3). In contrast, certain metabolites such as eicosatrienoic acid and tyrosine were negatively associated with air pollution exposures in PD patients but postiviely associated in controls – e.g., eicosatrienoic acid in the 1-year PM_2.5_ (CTM) model (PD: log_2_FC=−0.2, 95% CI=−0.3, −6.3e-4; Control: log_2_FC = 0.2, 95% CI = 0.03, 0.5) and tyrosine within a 3-year CO (CALINE4) model (PD: log_2_FC=−0.08, 95% CI=−0.1, −0.02; Control: log_2_FC = 0.1, 95% CI = 7.7e-4, 0.3).

Additional control-specific associations were observed between PM_2.5_ (LUR) exposure and inflammation-related lipids, with prostaglandin, linoleic acid, and epoxyoctadecadienoic acid (EpODE) all showing consistent negative association across multiple exposure averages (Supplementary Fig. 4; Supplementary Data 1). Effect estimates for these metabolites were similar in magnitude across 1-, 3-, and 5-year averages, suggesting stable, longer-term metabolic responses to fine particulate exposure.

### Pathway enrichment analysis

Inflammation-, lipid-, and amino acid-related pathways were enriched within MWAS-identified features that contained ≥5 metabolites and showed pathway overrepresentation at p < 0.05 (Supplementary Fig. 5; Supplementary Data 2). In PD patients, 15 pathways met this criterion. For PM_2.5_ (CTM), enriched pathways included lipid metabolism (e.g., butanoate metabolism, fatty acid activation, and de novo fatty acid biosynthesis) and amino acid metabolism (e.g., alanine/aspartate metabolism and alanine/aspartate/glutamate metabolism). For CO (CALINE4), tyrosine metabolism was enriched. For PM_2.5_ (LUR), enriched pathways included fatty acid metabolism, vitamin A (retinol) metabolism, and glycine/serine/alanine/threonine metabolism.

In controls, 15 pathways were enriched, primarily when the PM_2.5_ (LUR) was used as the exposure measure. These included inflammation-related pathways (e.g., arachidonic acid metabolism, linoleic acid metabolism, and prostaglandin formation from arachidonic acid).

## DISCUSSION

Using untargeted serum metabolomics with LC-HRMS, we identified 26 annotatable metabolites and 23 metabolic pathways associated with air pollution, particularly TRAP (measured by CO and NO_2_) and also PM_2.5_, in PD patients and in controls. The metabolic profiles we observed in controls aligned well with serum and plasma findings from 22 previous air pollution metabolomics studies (Figs. 2–3), supporting the validity of our approach and the comparability in air pollution exposure measures across these studies. Findings in controls help delineate general metabolic responses to air pollution from disease-modified responses seen in PD patients. While inflammatory lipid pathways were prominent in controls, PD patients exhibited additional alterations in amino acid, mitochondrial, and dopamine-related pathways. As summarized in Fig. 4, our findings suggest that air pollution induses systemic inflammatory lipid remodeling broadly, while PD patients demonstrate distinct metabolic vulnerability consistent with mitochondrial dysfunction and impaired dopaminergic metabolism.

### Shared metabolic responses to air pollution in controls

#### Inflammation-related lipid metabolism.

We identified disruptions in inflammation-related lipid metabolism with air pollution exposure in both controls and patients. PM_2.5_ and TRAP represent a complex mixture of pollutants, including PAHs,^21,22^ which can enter the body via inhalation and reach the bloodstream, contributing to systemic inflammation.^23–25^ These pollutants also generate reactive oxygen species (ROS),^26^ which target cell membranes, induce lipid peroxidation, and release polyunsaturated fatty acids (PUFAs), such as linoleic acid and arachidonic acid.^27^ The multiple carbon-carbon double bonds in these PUFAs make them highly susceptible to oxidation by oxygenase.^28^ In our study, anti-inflammatory linoleic acid decreased, while pro-inflammatory arachidonic acid increased with air pollution exposure in controls, aligning with findings from previous studies.^3,29–34^ Linoleic acid and arachidonic acid serve as substrates for lipoxygenase enzymes expressed by immune cells, leading to the production of major inflammatory mediators, including prostaglandins and leukotrienes.^35^ Identification of alterations in linoleic acid metabolism, arachidonic acid metabolism, and prostaglandin formation pathways in controls further suggests that air pollution disrupts inflammatory signaling and the antioxidant-oxidant balance.^3,31,33,36–39^ Importantly, these inflammatory pathways were also enriched in previous air pollution-metabolomics studies across diverse population, including pregnant women, children, and young adults, suggesting that inflammation likely represents a typical metabolic response to air pollution.

### PD-specific metabolic alterations

In contrast to controls, PD patients exhibited a distinct metabolic profile with air pollution exposure, characterized broader lipid and amino acid metabolism and less apparent inflammatory lipid signals. This shift likely reflects the disease context: PD patients already have baseline systemic and central inflammation, which may attenuate the additional inflammatory signal from air pollution. Instead, the metabolic response in patients was dominated by pathways related to mitochondrial dysfunction and dopamine synthesis. These patient-specific patterns may reflect disease-related susceptibility, disruptions in homeostasis, or compensatory responses, and are consistent with mechanisms previously implicated in PD pathogenesis and progression.^40–42^

#### Pro-inflammatory lipid shift and neuroinflammation.

Although inflammatory lipid signals were less prominent in PD patients than in controls, leukotriene was positively associated with air pollution exposure in patients, suggesting a shift toward a pro-inflammatory lipid profile. Evidence for leukotriene involvement in PD is more limited than in Alzheimer’s disease,^43^ but rodent models and mechanistic studies suggest that leukotrienes trigger microglia and astrocyte activation, leading to neuroinflammation and oxidative stress.^44,45^ This pro-inflammatory shift may also explain the negative associations of eicosatrienoic acid and HDHA with air pollution exposure in patients. Eicosatrienoic acid is preferentially converted to arachidonic acid, promoting leukotriene production instead of supporting DHA-related pathways (Fig. 4). Despite their neuroprotective roles as nutrients and membrane components, eicosatrienoic acid and DHA have limited bioavailability, as they are primarily bound to albumin or lipoproteins,^46^ leaving minimal free fatty acids in serum.^47^ Instead, they act as key regulators of metabolic and inflammatory pathways,^48^ as the free fatty acids detected in our untargeted MWAS are likely generated through phospholipase-mediated signaling.

#### TCA cycle disruption and mitochondrial dysfunction.

In PD patients, alanine, aspartate, and glutamate were negatively associated with air pollution exposure. These amino acids serve as key intermediates in the mitochondrial tricarboxylic acid (TCA) cycle: alanine is converted to pyruvate, aspartate feeds into oxaloacetate, and glumate enters the cycle via -ketoglutarate (Fig. 4). Their simultaneous depletion with higher air pollution exposure may impair ATP production and increase reliance on less efficient energy sources like fatty acid oxidation, exacervating mitochondrial dysfunction.

#### Tyrosine depletion and dopamine synthesis.

Tyrosine metabolism was enriched in PD patients, with tyrosine negatively associated with air pollution exposure. Tyrosine is converted into L-DOPA by tyrosine hydroxylase and subsequently decarboxylated into dopamine (Fig. 4), the neurotransmitter essential for controlling movement and one that is progressively lost in PD. The depletion of this pathway not only further disrupts TCA cycle activity but also directly reduces dopamine availability. Lower tyrosine levels have also been linked to severe depressive symptoms in our PD patients,^49^ suggesting that air pollution-induced tyrosine reduction may contribute to disease phenotypes such as depression by impairing mitochondrial function and decreasing dopamine availability.

### Limitations

To date, our study is the first untargeted HRM study examining air pollution in the context of PD. However, several limitations should be considered. While lifestyle factors such as smoking and diet can influence metabolite levels, these factors would only confound our findings if they were associated with air pollution exposure. To address this, we assessed whether smoking status (never vs. ever) and diet (measured by HEI-2015) were linked to air pollution exposure and found no associations. We adjusted for several covariates (age, gender, race/ethnicity, and education) to reduce confounding, though residual bias cannot be ruled out. Despite these limitations, the overlap of our results with those of multiple other air pollution metabolomic studies conducted in diverse populations (e.g., children, young adults, pregnant women, and older adults) strengthens our confidence that the metabolic features identified in our study are robustly linked to air pollution.

Untargeted LC-HRMS detects a broad range of metabolites, but many features remain unidentified. Annotation is based on m/z and retention time parameters from large databases (e.g., HMDB) and feature correlation structures. While this approach provides high-confidence putative annotations, it is not definitive. Some features matched multiple possible compounds (one-to-many matching), while others (≥ 40%) had no match in current databases, adding uncertainty to feature annotation. As metabolomic reference libraries (e.g., HMDB, KEGG) continue to expand, we expect that a greater proportion of these features will be annotated in the future.

Finally, our metabolomic measurements were based on a single blood draw, providing only a snapshot in time. Future longitudinal studies will be more informative in determining whether the metabolites identified here are causally related to PD pathomechanisms or clinical progression, or instead serve as exposure-response biomarkers.

## CONCLUSIONS

Our untargeted metabolomics study provides insights into some of the molecular mechanisms linking air pollution (PM_2.5_ and TRAP measured by CO and NO_2_) to PD, highlighting metabolic features and pathways involved in inflammation, oxidative stress, and mitochondrial dysfunction. The consistency of metabolic disruptions we observed in controls with findings from previous air pollution metabolomics studies supports the validity of our results. These findings strengthen the biological plausibility for air pollution playing a role in PD and provide molecular-level evidence that complements traditional epidemiological findings. The potential of PM_2.5_ and TRAP to disrupt metabolic responses associated with PD underscores the need for longitudinal studies with repeated measures of air pollution exposure, metabolomics, and motor and non-motor symptoms to determine whether pollution-induced metabolic changes precede or coincide with disease onset or progression.

## Supplementary Material

Supplementary Files

This is a list of supplementary files associated with this preprint. Click to download.

- supplementarydata1.xlsx
- supplementarydata2.xlsx
- supplementarymaterial.docx

## Figures and Tables

**Table 1.** Characteristics of PEG study participants with metabolomics data.

	No. (%) of participants	
Characteristic	Controls (n = 271)	PD patients (n = 616)
**Age at diagnosis/interview**, mean (SD), y	66.0 (11.5)	66.9 (10.4)
**Age at sample collection**, mean (SD), y	66.4 (13.1)	69.9 (10.0)
**PD duration**, mean (SD), y	-	3.0 (2.6)
**Gender**		
Male	169 (62.4%)	390 (63.3%)
Female	102 (37.6%)	226 (36.7%)
**Race and ethnicity**		
White	234 (86.3%)	468 (76.0%)
Latino	2 (0.7%)	105 (17.0%)
Native American	23 (8.5%)	25 (4.1%)
Asian	3 (1.1%)	13 (2.1%)
African American	9 (3.3%)	5 (0.8%)
**Education**, mean (SD), y	14.5 (3.1)	13.8 (4.5)
**Smoking status**		
Never	129 (47.6%)	327 (53.1%)
Former	126 (46.5%)	267 (43.3%)
Current	16 (5.9%)	22 (3.6%)
**Study wave**		
PEG1	186 (68.6%)	275 (44.6%)
PEG2	85 (31.4%)	341 (55.4%)
**Year of sample collection**, mean (SD), y	2008.0 (6.5)	2008.8 (5.0)

## Data Availability

The metabolomics data used in this study are publicly available on Metabolomics Workbench (Project ID: PR001964, https://doi.org/10.21228/M8VD96).
